# Supplementary material for: Bacillus subtilis biofilm matrix components target seed oil bodies to promote growth and anti-fungal resistance in melon
Source: Nat Microbiol. 2022 Jun 6;7(7):1001–15. doi: 10.1038/s41564-022-01134-8 (PMC9246715; doi:10.1038/s41564-022-01134-8)
Supplement: Supplementary file 1 — Supplementary Tables 1–8, references and source data files (list only). [file 41564_2022_1134_MOESM1_ESM.pdf]

nature portfolio

https://doi.org/10.1038/s41564-022-01134-8

Supplementary information

Bacillus subtilis biofilm matrix components target seed oil bodies to promote growth and anti-fungal resistance in melon

In the format provided by the authors and unedited

Bacillus subtilis biofilm matrix components target seed oil bodies to promote growth and anti-fungal resistance in melon

Berlanga-Clavero *et al.*

Supplementary information

Supplementary Tables S1 to S8

Supplementary references

Source Data Files - Figures 1 to 5

Image Source Data File - Figure 4

Source Data File - Extended Data Figure 1

Source Data File - Extended Data Figure 3

Source Data File - Extended Data Figure 4

Source Data File - Extended Data Figure 5

Table S1. Bacterial strains used in this study.

Strain	Genotype	Reference
B. subtilis NCIB 3610	Wild type, undomesticated strain	Laboratory collection
ΔtasA	*B. subtilis* NCIB 3610 *tasA::km*	Vlamakis et al., 2008[(1)]
JC81	*B. subtilis* NCIB 3610 (*tapA-sipW-tasA*)::spc *lacA*::(*tapA-sipW-tasA*$_{K68A,D69A}$)	Cámara-Almirón et al., 2020[(2)]
ΔtapA	*B. subtilis* NCIB 3610 (*tapA-sipW-tasA*)::spc *amyE*::(*tapA*$_{\Delta 13-234}$-*sipW-tasA*)	Chu et al., 2006[(3)]
Δ*bslA*	*B. subtilis* NCIB 3610 *bslA*::mls	Cámara-Almirón et al., 2020[(2)]
Δ*eps*	*B. subtilis* NCIB 3610 *(epsA-O)::tet*	Branda et al., 2006[(4)]
Δsrf	*B. subtilis* NCIB 3610 *srfAA*::mls	Branda et al., 2001[(5)]
Δpps	*B. subtilis* NCIB 3610 *pps*::Tn10(spc)	Tsuge et al, 1999[(6)]
Δpks	*B. subtilis* NCIB 3610 *pks*::spc	Straight et al., 2007[(7)]

Table S2. Primers used in Gene expression analysis by qRT-PCR.

Gene	Primers pairs	Sequence (5'→3')	Amplicon (bp)
ga20ox1	GA20ox1-F	GCATAGAGCAGTGGTGAACA	96
	GA20ox1-R	AACTCCCTTGGTGGCTTTAC	
cyp707a1	CYP707A1-F	GGCCATGAAAGCAAGGAAAG	104
	CYP707A1-R	CCATGAAAGATCCAAGGAGGT	
act7	ACT-F	CACTGGTATTGTGCTGGATTC	98
	ACT7-R	CAAGGTCCAAACGGAGAATG	

Table S3. MZmine2.37 with IIN settings used for preprocessing steps of LC-MS/MS data acquired in positive ion mode using the GNPS environment.

Process		Settings for For Q-Exactive data	Settings for For QTOF data
Mass detection	MS1	3.00E+04	2.0E+02
	MS2	5.00E+02	0
ADAP Chromatogram building	Min group size	4	10
	Group intensity threshold	1.70E+06	2.0E+02
	Min highest intensity	6.50E+04	5.0E+02
	m/z tolerance	0.002	0.002
Deconvolution [Local minimum search]	Chromatographic threshold (%)	83	90
m/z center calculation: AUTO	Search minimum in RT range (min)	0.03	0.05
m/z range MS2 scan pairing: 0.01Da	Min relative height (%)	1	1
RT range MS2 scan pairing: 0.15 min	Minimum absolute height	8.00E+04	5.0E+02
	Min ratio of peak top/edge	1	2
	Peak duration range (min)	0.0 - 3.0	0.03 – 3.0
Isotopic peak grouper	*m/z* tolerance	0.0015	0.002
	RT tolerance	0.07 min	0.1
	Max charge	3	2
Alignment [Join Aligner]	*m/z* tolerance	0.0015	0.002
	Weight for m/z	2	20
	Weight for RT	1	0.1
	RT tolerance	0.35 min	0.1 min
Duplicate peak filter	*m/z* tolerance	0.001	0.002

Filter Mode:NEW AVERAGE	RT tolerance	0.1 min	0.05 min
Gap filling [Peak finder (multithreaded)]	Intensity tolerance	5%	1%
	m/z tolerance	0.001	0.002
	RT tolerance	0.12 min	0.1 min
Peak filter	#Data points	3.0-1.0E+04	3.0-1.0E+04
Peak list rows filter	Min peaks in a row	5	5
	Reset peak number ID	Checked	Checked
metaCorrelate	RT tolerance	0.08	0.09
	Min height	5.00E+04	4.0E+02
	Noise level	3.00E+04	2.0E+02
	Min samples filter	Min samples in all abs = 2 rel = 0	Min samples in all abs = 2 rel = 0
		Min samples in group abs = 0 rel = 0	Min samples in group abs = 0 rel = 0
		Min %-intensity overlap: 50	Min %-intensity overlap: 50
		Exclude estimated features (gap-filled)	Exclude estimated features (gap-filled)
	Correlation grouping (checked)	Min data points: 5	Min data points: 5
		Min data points on edge: 2	Min data points on edge: 2
		Measure: Pearson	Measure: Pearson
		Min feature shape correlation: 65.0%	Min feature shape correlation: 65.0%
		Min total correlation: unchecked	Min total correlation: unchecked
	Feature height correlation (checked)	Min data points: 3	Min data points: 3
		Measure: Pearson	Measure: Pearson
		Min correlation: 65.0%	Min correlation: 65.0%
Ion identity networking	*m/z* tolerance	0.001	0.001
	Check	ONE FEATURE	ONE FEATURE
	Min height	1.00E+05	1.00E+05

	Annotation refinement (checked)	Min size (checked): 2	Min size (checked): 2
		Delete small network without major	Delete small network without major
		Delete networks without monomer	Delete networks without monomer
	Ion identity library	MS mode: POSITIVE	MS mode: POSITIVE
		Max charge: 2	Max charge: 2
		Max molecule/cluster: 2	Max molecule/cluster: 2
		Adducts: $[M+H]^+$, $[M+Na]^+$, $[M+NH4]^+$, $[M+2H]^{2+}$	Adducts: $[M+H]^+$, $[M+Na]^+$, $[M+NH4]^+$, $[M+2H]^{2+}$
		Modifications: $[M-H_2O]$, $[M-2H_2O]$	Modifications: $[M-H_2O]$, $[M-2H_2O]$
Ion identity networking refinement	Min size (checked)	2	2
	Delete small network without major	Checked	Checked
	Delete smaller networks: Link threshold (checked)	3	3
	Delete networks without monomer	Checked	Checked
Add ion identities to networks	*m/z* tolerance	0	0
	Min height	8.00E+04	8.00E+04
	Annotation refinement (checked)	Delete small networks without major (checked)	Delete small networks without major (checked)
		Delete smaller networks: Link threshold (checked): 3	Delete smaller networks: Link threshold (checked): 3
		Delete networks without monomer	Delete networks without monomer
	Ion identity library	MS mode: POSITIVE	MS mode: POSITIVE
		Max charge: 2	Max charge: 2

		Max molecules/cluster: 3	Max molecules/cluster: 3
		Adducts: $[M+H]^+$, $[M+Na]^+$, $[M+K]^+$,$[M+NH4]^+$, $[M+2H]^{2+}$, $[M+H+Na]^{2+}$, $[M-H+2Na]^+$,	Adducts: $[M+H]^+$, $[M+Na]^+$, $[M+K]^+$,$[M+NH4]^+$, $[M+2H]^{2+}$, $[M+H+Na]^{2+}$, $[M-H+2Na]^+$,
		Modifications: $[M-H_2O]$, $[M-2H_2O]$, [M-CO], $[M-CO_2]$, [M+HAc], [M+ACN]	Modifications: $[M-H_2O]$, $[M-2H_2O]$, [M-CO], $[M-CO_2]$, [M+HAc], [M+ACN]
Check all ion identities by MS/MS	*m/z* tolerance (MS2)	0.0015	0.0015
	Min height in MS2	1.00E+03	1.00E+03
	Check for multimers	Checked	Checked
	Check neutral losses (MS->MS2)	Checked, PRECURSOR	Checked, PRECURSOR
Export for/Submit to GNPS	Merge MS/MS	Select spectra to merge: same sample	Select spectra to merge: same sample
	Filter only with MS2 or annotation	*m/z* merge mode: weighted average (remove outliers)	*m/z* merge mode: weighted average (remove outliers)
		Intensity merge mode: sum intensities	Intensity merge mode: sum intensities
		Expected mass deviation: 0.001	Expected mass deviation: 0.001
		Cosine threshold: 70.0%	Cosine threshold: 70.0%
		Peak count threshold: 20.0%	Peak count threshold: 20.0%
		Isolation window offset: 0	Isolation window offset: 0
		Isolation window width: 1.5	Isolation window width: 1.5
Export for SIRIUS	Merge MS/MS	Select spectra to merge: same sample	Select spectra to merge: same sample

		m/z merge mode: weighted average (remove outliers)	*m/z* merge mode: weighted average (remove outliers)
		Intensity merge mode: sum intensities	Intensity merge mode: sum intensities
		Expected mass deviation: 0.001	Expected mass deviation: 0.001
		Cosine threshold: 70.0%	Cosine threshold: 70.0%
		Peak count threshold: 20.0%	Peak count threshold: 20.0%
		Isolation window offset: 0	Isolation window offset: 0
		Isolation window width: 1.5	Isolation window width: 1.5
	m/z tolerance	0.001	0.001
	Exclude empty MS/MS spectra	Checked	Checked
	Exclude multiple charge	Checked	Checked
	Exclude multimers	Checked	Checked

Table S4. Settings used for FBMN using the GNPS environment.

**Molecular networking**	
Data filtering	Removing all MS/MS fragment ions within +/- 17 Da of the precursor m/z
Window filter	Top 6 fragment ions in the +/- 50 Da window throughout the spectrum
precursor ion mass tolerance	0.02 Da
MS/MS fragment ion tolerance	0.02 Da
cosine score	0.7
Minimum matched peaks	6
maximum size of a molecular family	100
**Library search**	
Score Threshold	0.6
Minimum matched peaks	5

Table S5. HPLC-ESI-MS/MS analysis of co-eluting bands obtained in the pull-down assay between TasA and a seed protein extract.

Protein FDR	Accession	Description	q-value	PEP Score	Coverage [%]	# AAs	MW [kDa]
High	A0A1S3C9Y1	Oleosin	0	65.897	35	182	19.5
High	A0A1S3B8E2	Oleosin	0	35.099	18	176	19
High	A0A1S3BXD3	Oleosin	0	30.473	20	142	15

Table S6. Average RPKM (reads per kilobase per million) counts of a selection of induced genes in leaves of plants from fengycin-treated seeds 0 hours (before the challenge with *B. cinerea* and 48 hours after the challenge.

		**Control**		**Fengycin**	
**ID**	**Description**	**0h**	**48h**	**0h**	**48h**
MELO3C014485	sugar transport protein 13	96	1075	3256	3473
MELO3C003015	Allene oxide cyclase	133	2845	36985	83705
MELO3C004455	blue copper protein-like	7	219	328	753
MELO3C014620	Glycosyltransferase	16	333	979	1486
MELO3C017499	1-deoxy-D-xylulose-5-phosphate synthase	211	1473	7668	6421
MELO3C029299	12-oxophytodienoate reductase family protein	1189	3653	11733	12813
MELO3C009160	trihelix transcription factor GT-3b	20	268	802	2148
MELO3C014239	Aquaporin PIP2	135	668	3371	4829
MELO3C002598	E3 ubiquitin-protein ligase RMA1H1-like	339	1583	7543	7192
MELO3C018463	CBS domain-containing protein CBSX1, chloroplastic	14	155	3006	5753
MELO3C026019	Abscisic acid receptor	703	1707	1405	839
MELO3C022678	Protein TIFY 10B	200	732	6514	8195
MELO3C015780	Calcium-transporting ATPase	64	314	2606	4396
MELO3C007431	alkaline ceramidase 3	724	3396	1125	1705
MELO3C012630	cysteine-rich and transmembrane domain-containing protein A-like	120	395	2956	4144
MELO3C017151	protein NRT1/ PTR FAMILY 2.11-like	721	2961	13668	22129
MELO3C003567	Glycosyltransferase	1072	2497	14918	21020
MELO3C015514	12-oxophytodienoate reductase 3	116	346	1814	1705
MELO3C024499	Threonine dehydratase	328	2309	25591	30235
MELO3C013916	ethylene-responsive transcription factor 2	668	1516	10405	5418
MELO3C015881	Ankyrin repeat protein	147	477	2235	2178
MELO3C023587	Protein BPS1 chloroplastic	130	382	2255	2940
MELO3C009170	vacuolar cation/proton exchanger 3-like	1044	2172	1156	1398
MELO3C010910	Allene oxide synthase	3990	8544	19333	31388
MELO3C014304	chlorophyllase-1-like	42	165	10351	13675
MELO3C008331	ethylene-responsive transcription factor ERF113-like	612	1255	4202	5603

Table S7. Seed species tested for growth promotion activity after treatment with fengycin.

**Plant species**	**Family**	**Structure**		**Growth promotion activity**
Arabidopsis thaliana	*Brassicaceae*	Embryo surrounded by a single cell layer of endosperm	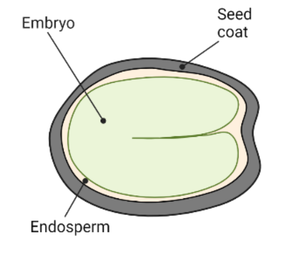	No
Solanum lycopersicum	*Solanaceae*	Embryo surrounded by abundant endosperm	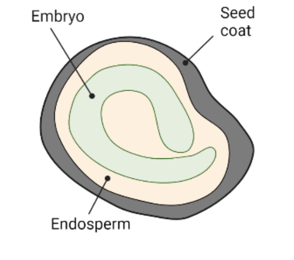	No
Cucumis sativus	*Cucurbitaceae*	Embryo surrounded by a perisperm/endosperm envelope	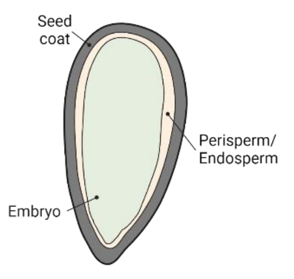	Small tendence
Cucumis melo	*Cucurbitaceae*			Yes
Cucurbita pepo	*Cucurbitaceae*			No
Glycine max	*Fabaceae*	Non endospermic seed, embryo enclosed by the seed coat	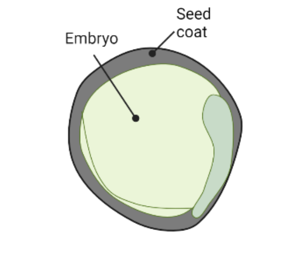	Yes
Zea mays	*Poaceae*	Apical embryo and a starchy endosperm surrounded by the aleurone layer	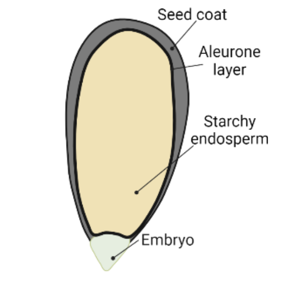	No
Tritium spp	*Poaceae*			No

Images adapted from Leubner G (2021). The Seed Biology Place - http://www.seedbiology.eu

Table S8. Named features along the manuscript and their parameters for being identified within the Sumner et al., 2007 framework.

Proposed Compound Name	IonMode	Adduct	observed m/z	calculated m/z	ppm error	Mass Diff. m/z	Level of identification
POPC	Positive	M+H	760.5822	760.586	4.97535		2
Analog to TAG (18:1/18:2/18:2)	Positive	M+NH4	898.76	880.75		-18.01	3
PC (0:0/16:0)	Positive	M+H	522.3553	495.332	2.45377		2
L-Tryptophan	Positive	M+H-NH3	188.0709	188.0709	4.78688		2
Glutathione, oxidized	Positive	M+H	613.1589	613.151	12.8411		2
Dilinolenin (9c,12c,15c)	Positive	M+H-H2O	595.4672	595.4672	9.73737		2
3,4-Dihydroxycinnamic acid	Positive	[M+H]	181.0497	181.0497	1.68559		2
Monolinolenin (9c,12c,15c)	Positive	M+H-H2O	335.256	335.256	5.91676		2
Analog to Mangiferin	Positive	M+H	433.1132	423.09		-10.0232	3
Analog to 2-O-Rhamnosylvitexin	Positive	M+H	595.1657	579.171		-15.9947	3
Analog to TAG (16:0/18:1/18:2)	Positive	M+NH4	790.65	874.78		84.13	3
Analog toTAG (17:1/18:1/18:2)	Positive	M+NH4	914.78	886.78		-28	3
L-glutathione, reduced	Positive	M+H	308.09	308.09	5		2
16:0 Lyso PE	Positive	M+H	496.34	496.34	8		2

Supplementary references

1. Vlamakis, H., Aguilar, C., Losick, R. & Kolter, R. Control of cell fate by the formation of an architecturally complex bacterial community. *Genes Dev*. **22**, 945–953 (2008).

2. Cámara-Almirón, J. *et al.* Dual functionality of the amyloid protein TasA in Bacillus physiology and fitness on the phylloplane. *Nat. Commun.* **11**, 1–21 (2020).

3. Chu, F., Kearns, D. B., Branda, S. S., Kolter, R. & Losick, R. Targets of the master regulator of biofilm formation in *Bacillus subtilis*. *Mol. Microbiol.* **59**, 1216–1228 (2006).

4. Branda, S. S., Chu, F., Kearns, D. B., Losick, R. & Kolter, R. A major protein component of the *Bacillus subtilis* biofilm matrix. *Mol. Microbiol*. **59**, 1229–1238 (2006).

5. Branda, S. S., González-Pastor, J. E., Ben-Yehuda, S., Losick, R. & Kolter, R. Fruiting body formation by *Bacillus subtilis*. Proc. Natl. Acad. Sci. U. S. A. **98**, 11621–6 (2001).

6. Tsuge, K., Ano, T., Hirai, M., Nakamura, Y. & Shoda, M. The genes *degQ*, *pps*, and *lpa-8* (sfp) are responsible for conversion of *Bacillus subtilis* 168 to plipastatin production. *Antimicrob. Agents Chemother.* **43**, 2183–92 (1999).

7. Straight, P. D., Fischbach, M. A., Walsh, C. T., Rudner, D. Z. & Kolter, R. A singular enzymatic megacomplex from *Bacillus subtilis*. *Proc. Natl. Acad. Sci. U. S. A.* **104**, 305–10 (2007).
