# Supplementary material for: Bacillus subtilis biofilm matrix components target seed oil bodies to promote growth and anti-fungal resistance in melon
Source: Nat Microbiol. 2022 Jun 6;7(7):1001–15. doi: 10.1038/s41564-022-01134-8 (PMC9246715; doi:10.1038/s41564-022-01134-8)
Supplement: Supplementary file 2 — Reporting Summary [file 41564_2022_1134_MOESM2_ESM.pdf]

nature portfolio

Corresponding author(s): Diego Romero

Last updated by author(s): Apr 20, 2022

Reporting Summary

Nature Portfolio wishes to improve the reproducibility of the work that we publish. This form provides structure for consistency and transparency in reporting. For further information on Nature Portfolio policies, see our Editorial Policies and the Editorial Policy Checklist.

Statistics

For all statistical analyses, confirm that the following items are present in the figure legend, table legend, main text, or Methods section.

n/a	Confirmed	
☐	☒	The exact sample size (*n*) for each experimental group/condition, given as a discrete number and unit of measurement
☐	☒	A statement on whether measurements were taken from distinct samples or whether the same sample was measured repeatedly
☐	☒	The statistical test(s) used AND whether they are one- or two-sided<br>*Only common tests should be described solely by name; describe more complex techniques in the Methods section.*
☒	☐	A description of all covariates tested
☒	☐	A description of any assumptions or corrections, such as tests of normality and adjustment for multiple comparisons
☐	☒	A full description of the statistical parameters including central tendency (e.g. means) or other basic estimates (e.g. regression coefficient) AND variation (e.g. standard deviation) or associated estimates of uncertainty (e.g. confidence intervals)
☐	☒	For null hypothesis testing, the test statistic (e.g. *F*, *t*, *r*) with confidence intervals, effect sizes, degrees of freedom and *P* value noted<br>*Give P values as exact values whenever suitable.*
☒	☐	For Bayesian analysis, information on the choice of priors and Markov chain Monte Carlo settings
☒	☐	For hierarchical and complex designs, identification of the appropriate level for tests and full reporting of outcomes
☒	☐	Estimates of effect sizes (e.g. Cohen's *d*, Pearson's *r*), indicating how they were calculated

Our web collection on statistics for biologists contains articles on many of the points above.

Software and code

Policy information about availability of computer code

Data collection	RNA-seq data were collected using NextSeq System Suite v2.2.0.<br>Confocal microscopy images were taken using Leica Application SuiteAdvance Fluorescence v2.7.3.9723.<br>Electron microscopy images were taken using TIA FEI Imaging Software v4.14.<br>Metabolomics data were obtained with the commercial software of Thermo Scientific for UPLC systems.<br>HPLC-ESI-MS/MS data corresponding to the identification of proteins in gel cut bands was acquired using ProteinScape 3 software from Bruker coupled to Mascot v3.1 (Matrix Science).
Data analysis	For statistics and representation of all experimental data, analysis were done using GraphPad Prism v.6.01.<br><br>Raw reads were preprocessed by NextSeq System Suite v2.2.0. using specific NGS technology configuration parameters.<br>Clean reads of the BAM files were aligned and annotated using the Cucumis melo genome (v4.0) as the reference by Bowtie2; these data were then sorted and indexed using SAMtools v1.48411074. Uniquely localized reads were used to calculate the read number values for each gene by Sam2counts (https://github.com/vsbuffalo/sam2counts). Differentially expressed genes (DEGs) in the treatment samples were analyzed by DEgenes Hunter, which provides a combined p value calculated based on the Fisher method using nominal p values provided by edgeRand DEseq2. This combined p value was adjusted by the Benjamini-Hochberg (BH) procedure (false discovery rate approach) and used to rank all obtained differentially expressed genes.<br>A heatmap and DEG clustering were generated using ComplexHeatmap in R Studio and Kobas 2.0.<br>DEGs annotated using the Cucumis melo genome were processed to identify the Gene Ontology functional categories using sma3s and TopGo software.<br>KEGG pathways and enrichment were estimated using Bioconductor packages (Bioconductor.org) GGplot2, ClusterProfiler, DOSE, and EnrichPlot in R Studio.<br><br>For LC-MS/MS data analysis, raw spectra were converted to .mzXML files using MSconvert (ProteoWizard). MS1 and MS/MS feature extraction was performed with Mzmine2.30. For molecular networking and spectrum library matching the .mgf file was uploaded to GNPS

(gnps.ucsd.edu). Molecular networks were visualized with Cytoscape v. 3.4.

For CLSM data analysis, image processing was performed using Leica LAS AF (LCS Lite, Leica Microsystems) and ImageJ 1.51s.

For manuscripts utilizing custom algorithms or software that are central to the research but not yet described in published literature, software must be made available to editors and reviewers. We strongly encourage code deposition in a community repository (e.g. GitHub). See the Nature Portfolio guidelines for submitting code & software for further information.

Data

Policy information about availability of data

All manuscripts must include a data availability statement. This statement should provide the following information, where applicable:

- Accession codes, unique identifiers, or web links for publicly available datasets
- A description of any restrictions on data availability
- For clinical datasets or third party data, please ensure that the statement adheres to our policy

The RNA-seq data were deposited in the GEO database under the reference GSE175611.

Metabolomics data are deposited at https://massive.ucsd.edu/ with the identifiers MSV000084674 (data from radicles from seeds bacterized with Bacillus subtilis subsp subtilis NCBI 3610 and mutant strains; https://massive.ucsd.edu/ProteoSAFe/dataset.jsp?task=b2e3636335d24c2da72634b7c6c8b63f), MSV000084278 (data from radicles from the seeds treated with fengycin and TasA; https://massive.ucsd.edu/ProteoSAFe/dataset.jsp?task=fc05e1b986cb4073b56d9f9a044f0a2f), MSV000086360 (data from adult plants grown from control seeds or seeds bacterized with Bacillus subtilis subsp subtilis NCBI 3610; https://massive.ucsd.edu/ProteoSAFe/dataset.jsp?task=1090c82889a04e4ba9be1058a8a5d6db) and MSV000088139 (data from seeds and seedlings treated with fengycin, TasA or bacteria; https://massive.ucsd.edu/ProteoSAFe/dataset.jsp?task=72bb762b7b8d45da8d3c5c9005a425d8).

Cucurbit genome databases used for mass spectrometry analysis of protein bands were UniProtKB/TrEMBL TrEMBL Cucumis melo, v 2017.10.25.

Field-specific reporting

Please select the one below that is the best fit for your research. If you are not sure, read the appropriate sections before making your selection.

☒ Life sciences ☐ Behavioural & social sciences ☐ Ecological, evolutionary & environmental sciences

For a reference copy of the document with all sections, see nature.com/documents/nr-reporting-summary-flat.pdf

Life sciences study design

All studies must disclose on these points even when the disclosure is negative.

Sample size	Sample-size calculation was not initially performed but a sufficient number of replicates were taken, prior experience with these types of data, and analysis of the current data, which indicates the reproducibility of the data. The number of replicates is sufficient in order to get statistically confident results, as demonstrated in the manuscript. In all the experiments performed, standard deviation and standard error were calculated and the corresponding statistical tests were performed when needed.
Data exclusions	No data were excluded from the study.
Replication	At least three replicates were taken in all the experiments performed. In all the cases the experiments were successfully replicated
Randomization	Randomization is not relevant in this case as our study is mainly focused in a plant-bacteria interaction.
Blinding	Blinding is not relevant in this case as our study is mainly focused in a plant-bacteria interaction.

Reporting for specific materials, systems and methods

We require information from authors about some types of materials, experimental systems and methods used in many studies. Here, indicate whether each material, system or method listed is relevant to your study. If you are not sure if a list item applies to your research, read the appropriate section before selecting a response.

Materials & experimental systems

n/a	Involved in the study
☐	☒ Antibodies
☒	☐ Eukaryotic cell lines
☒	☐ Palaeontology and archaeology
☒	☐ Animals and other organisms
☒	☐ Human research participants
☒	☐ Clinical data
☒	☐ Dual use research of concern

Methods

n/a	Involved in the study
☒	☐ ChIP-seq
☒	☐ Flow cytometry
☒	☐ MRI-based neuroimaging

Antibodies

Antibodies used

The anti-TasA antibody used for immunodetection studies in this work was kindly provided by professor Adam Driks (Stover and Driks, 1999) and it consists in a polyclonal antiserum obtained from blood 23 days after injecting the protein into rabbbit.
The secondary antibody used in western blot was commercially available at Bio-Rad (Goat Anti-Rabbit IgG (H + L)-HRP Conjugate cat. no. 1706515). The immunogold-conjugated secondary antibody used was coomercially available at BBI solutions (EM Grade 20nm Goat anti-Rabbit Conjugate cat. no. EM.GAR20/1).

Validation

The anti-TasA primary antibody was validated elsewhere in the literature (Stover and Driks, 1999).
The Goat Anti-Rabbit IgG (H + L)-HRP Conjugate (cat. no. 1706515) secondary antibody from BioRad was validated by the supplier as follows: "Specific for rabbit IgG, heavy and light chain. The cross-reactivities of anti-rabbit IgG antibody are tested in an ELISA. Minimum cross-reactivity to human IgG. 1:2,000-1:5,000 dilution can be used (Coligan, J., 1997)".
