# Supplementary material for: Bacillus subtilis biofilm matrix components target seed oil bodies to promote growth and anti-fungal resistance in melon
Source: Nat Microbiol. 2022 Jun 6;7(7):1001–15. doi: 10.1038/s41564-022-01134-8 (PMC9246715; doi:10.1038/s41564-022-01134-8)

Source Data File Figure 4

Pull down assay. SDS-PAGE Protein gel, Commasie Brilliant Blue staining (FT: Flow trough, W: wash, E: elution). Units of the molecular weight marker are kDa.

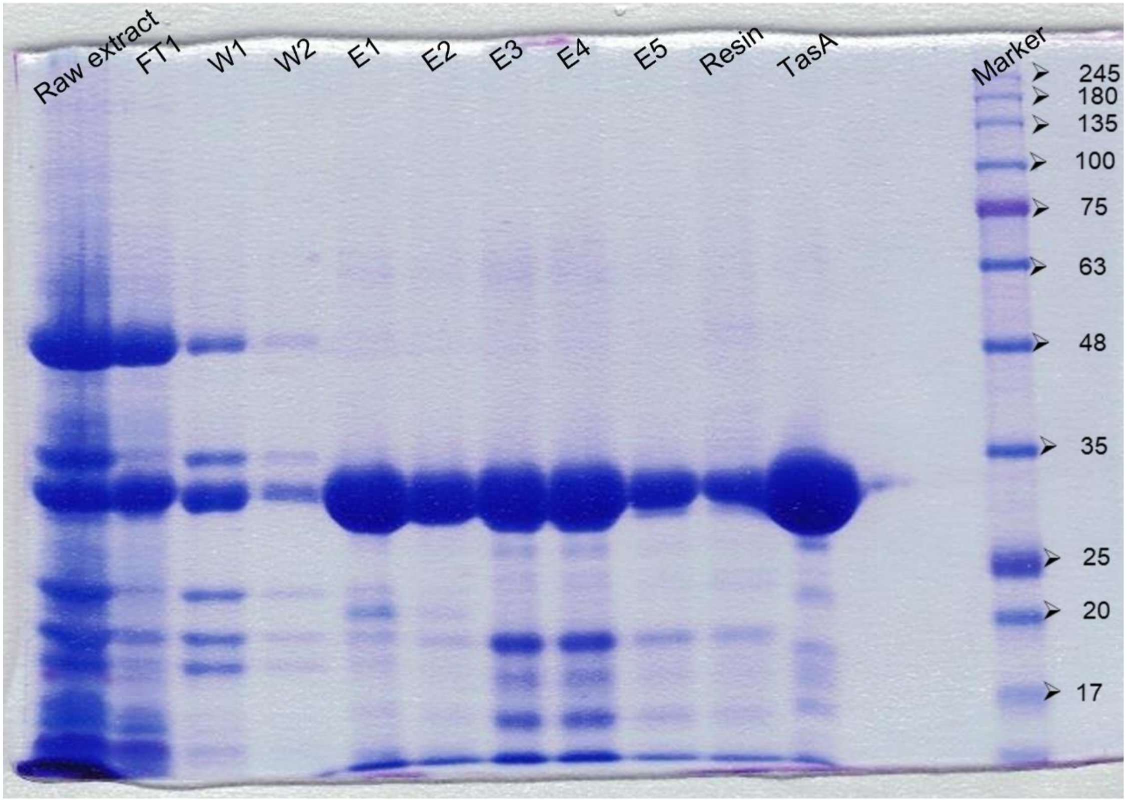

Pull down assay. SDS-PAGE Protein gel, Commasie Brilliant Blue staining. Negative control: no TasA added to the resin.

(FT: Flow trough, W: wash, E: elution). Units of the molecular weight marker are kDa.

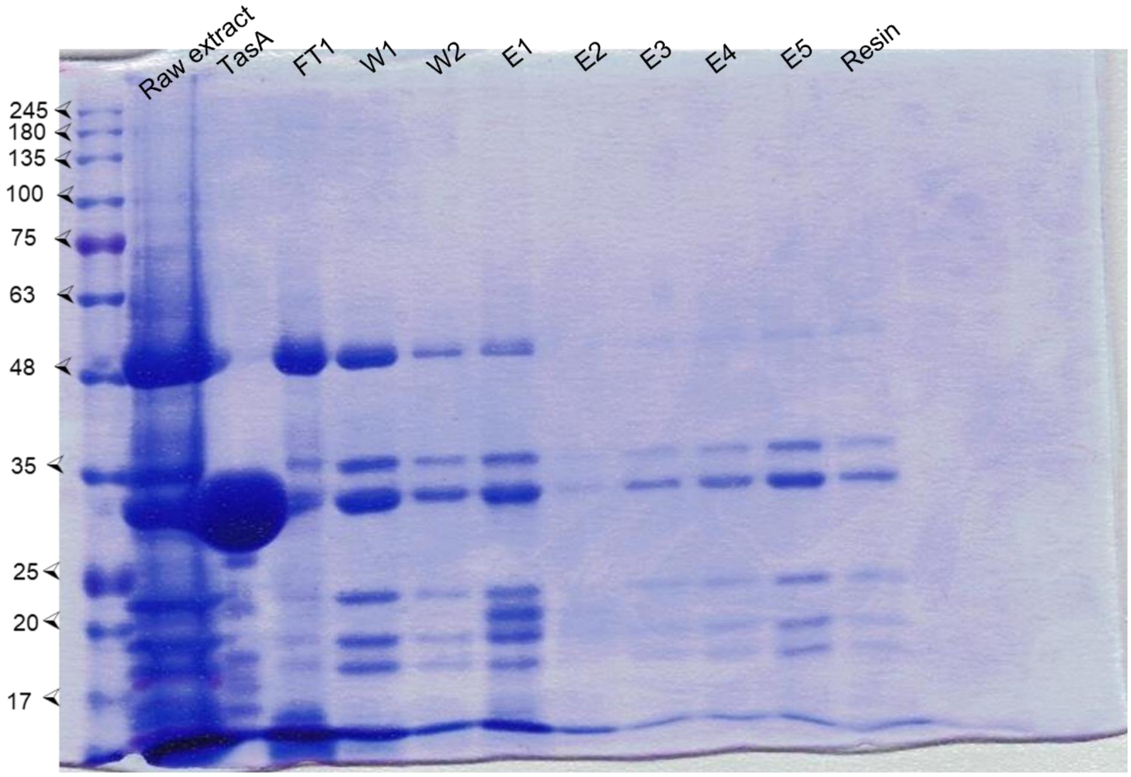

Far Western Blot assay. SDS-PAGE Protein gel, Commasie Brilliant Blue staining. Units of the molecular weight marker are kDa.

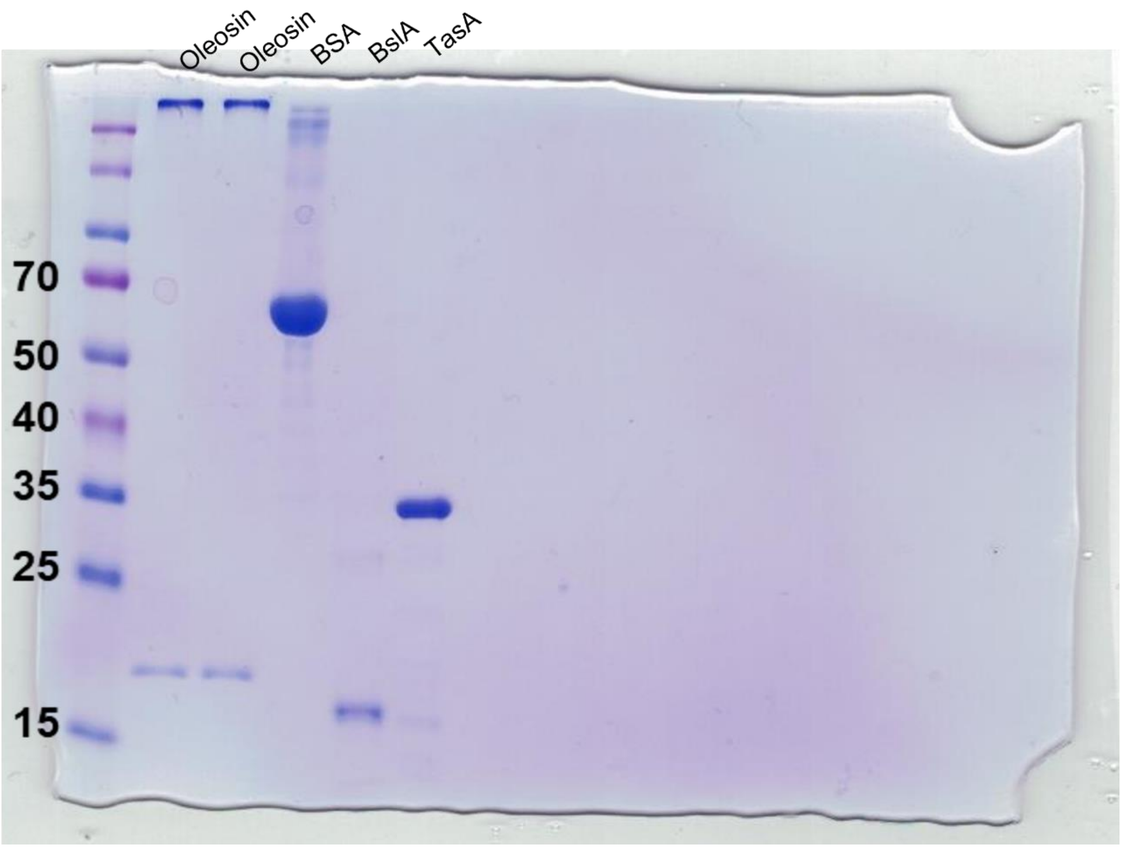

Far Western Blot assay. SDS-PAGE Protein gel transferred to PVDF membrane. Western Blot using an anti-TasA antibody (1:20.000).

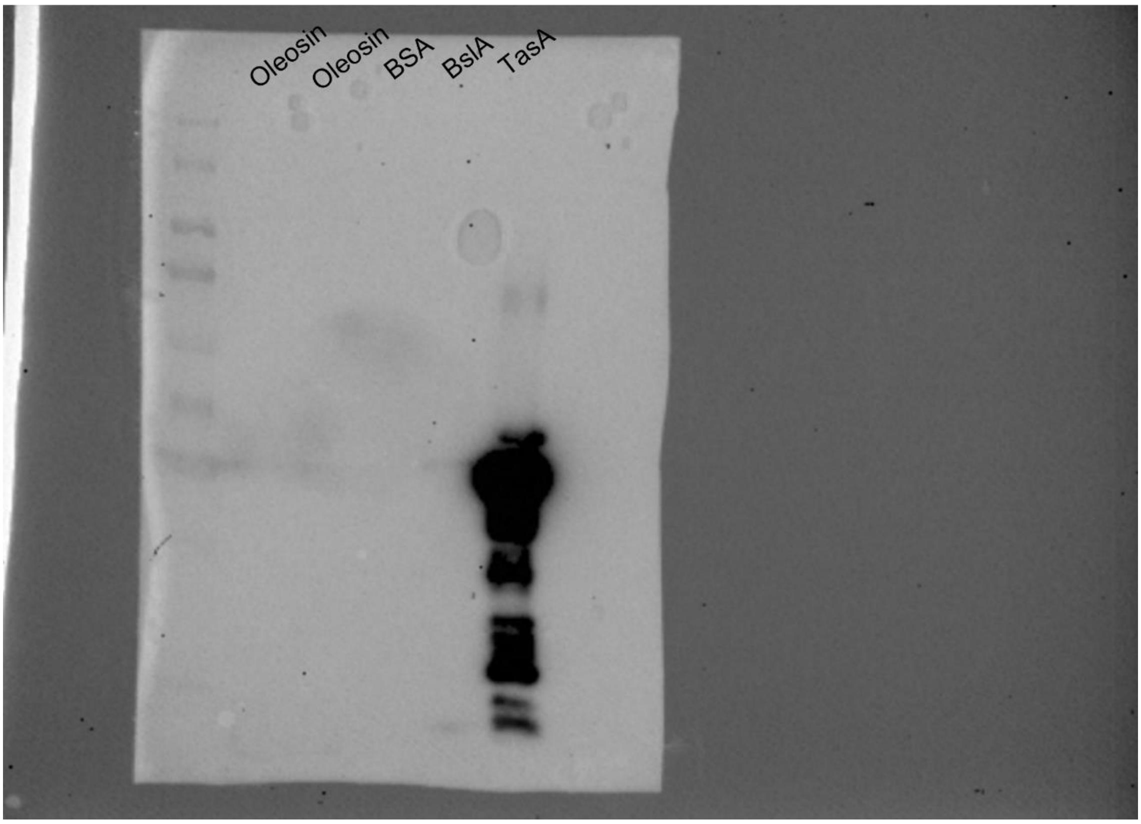

Far Western Blot assay. SDS-PAGE Protein gel transferred to PVDF membrane. Western Blot using an anti-TasA antibody (1:20.000) after renaturing of the proteins and membrane incubation with TasA as a bait protein.

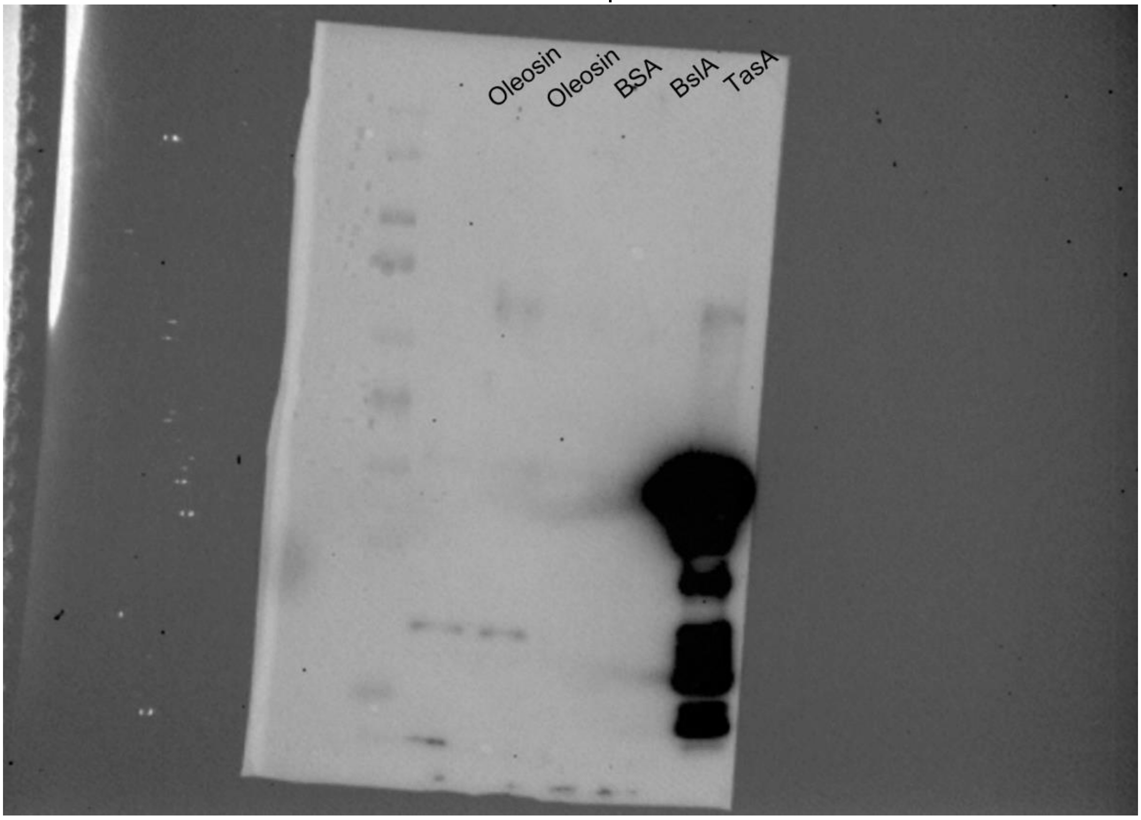

Supplement: Source Data Fig. 4 — Unprocessed gels and western blots. [file 41564_2022_1134_MOESM8_ESM.pdf]
